# A comparative study of psychotic and affective symptoms in Rwandan and Kenyan students

**DOI:** 10.1017/S2045796016001074

**Published:** 2017-01-26

**Authors:** A. Owoso, S. Jansen, D. M. Ndetei, A. Musau, V. N. Mutiso, C. Mudenge, A. Ngirababyeyi, A. Gasovya, D. Mamah

**Affiliations:** 1 Department of Psychiatry, Washington University School of Medicine, St. Louis, MO, USA; 2 Center for Mental Health, University of Rwanda College of Medicine and Health Sciences, Kigali, Rwanda; 3 Africa Mental Health Foundation, Nairobi, Kenya; 4 Department of Psychiatry, University of Nairobi, Nairobi, Kenya; 5 University Teaching Hospital of Butare, Rwanda

**Keywords:** Africa, adolescents, high-risk, war, WERCAP

## Abstract

**Aims.:**

War and conflict are known to adversely affect mental health, although their effects on risk symptoms for psychosis development in youth in various parts of the world are unclear. The Rwandan genocide of 1994 and Civil War had widespread effects on the population. Despite this, there has been no significant research on psychosis risk in Rwanda. Our goal in the present study was to investigate the potential effects of genocide and war in two ways: by comparing Rwandan youth born before and after the genocide; and by comparing Rwandan and Kenyan adolescents of similar age.

**Methods.:**

A total of 2255 Rwandan students and 2800 Kenyan students were administered the Washington Early Recognition Center Affectivity and Psychosis (WERCAP) Screen. Prevalence, frequency and functional impairment related to affective and psychosis-risk symptoms were compared across groups using univariate and multivariate statistics.

**Results.:**

Rwandan students born before the end of the genocide and war in 1994 experienced higher psychotic and affective symptom load (*p*’s < 0.001) with more functional impairment compared with younger Rwandans. 5.35% of older Rwandan students met threshold for clinical high-risk of psychosis by the WERCAP Screen compared with 3.19% of younger Rwandans (*χ*^2^ = 5.36; *p* = 0.02). Symptom severity comparisons showed significant (*p* < 0.001) group effects between Rwandan and Kenyan secondary school students on affective and psychotic symptom domains with Rwandans having higher symptom burden compared with Kenyans. Rwandan female students also had higher rates of psychotic symptoms compared with their male counterparts – a unique finding not observed in the Kenyan sample.

**Conclusions.:**

These results suggest extreme conflict and disruption to country from genocide and war can influence the presence and severity of psychopathology in youth decades after initial traumatic events.

## Introduction

War has long been understood to have an influence on mental health outcomes in populations (Murthy & Lakshminarayana, 2006); impacts of the stress from war have been studied in multiple regions, including the Balkans (Jovanovic *et al.* 2010; Priebe *et al.* 2010*b*), Afghanistan (Cardozo *et al.* 2004; Scholte *et al.* 2004), South Asia (Siriwardhana *et al.* 2015), sub-Saharan Africa (Mugisha *et al.* 2015) and the Middle East (Neria *et al.* 2010). While all of society bears a burden from conflict, children are particularly vulnerable (Ghosh *et al.* 2004). In 2014, UNICEF reported that 230 million children live in areas of armed conflict; this number includes victims and witnesses of violence, child soldiers, and displaced children, among others (UNICEF, 2014).

Many studies have investigated mental health impacts of conflict on youth (Panter-Brick *et al.* 2009; Schiff *et al.* 2012; Okello *et al.* 2014; Kangaslampi *et al.* 2015), though the majority have explored affective and anxiety-related pathologies – particularly depression and posttraumatic stress disorder (PTSD) (Panter-Brick *et al.* 2009; Kimhi *et al.* 2010; Okello *et al.* 2014; Tol *et al.* 2014; Kangaslampi *et al.* 2015). A series of studies (Silove *et al*. 2008, 2014; Soosay *et al*. 2012) explored relationships between trauma exposure, PTSD symptomatology and psychotic-like experiences (PLEs) in post-conflict Timor. In one study, 12% screened positive for significant PLEs (Soosay *et al.* 2012). However, these studies focused primarily on adults. The dearth of research on conflict exposure and psychosis in youth is significant because of the connections between early-life stress and psychosis development. This elevated risk has been observed in child abuse victims (Choi *et al.* 2015), various forms of maltreatment (Read *et al.* 2008) and other childhood traumas (Hammersley *et al.* 2008; Bentall *et al.* 2012). Research has shown early life trauma and major adversities before age 18 are predictive of later psychosis and can increase the risk of developing psychotic illness by up to threefold later in life (Varese *et al.* 2012; Russo *et al.* 2014).

One of the most devastating recent conflicts was the Rwandan Civil War, beginning in 1990 and culminating in the genocide targeted towards the Tutsi people in 1994. During the genocidal period alone, an estimated 500 000–1 000 000 Rwandans were killed, with many more affected by displacement, abuse, torture, rape, forced combat or directly witnessing atrocities (Des Forges, 1999; Rieder & Elbert, 2013). The effects of this conflict were also felt by other countries due to refugee migration and proxy wars (Prunier, 2009). The impacts of the war on mental health have been studied in Uganda. Research there has described depressive and anxiety symptoms, behavioural disturbances and other psychological impacts on child soldiers (Klasen *et al.* 2010; Moscardino *et al.* 2012) and abducted individuals (Okello *et al.* 2007). Another study of child soldiers also showed connections between war experience, PTSD and psychosis (Amone-P'Olak *et al.* 2014); while relationships between PTSD and PLEs were similar to those seen in Timor, investigation was not extended to the general youth population. Though there has been some work on the effects of the war on the Rwandan population, much of it has focused on affective symptoms in adults (e.g. Lacasse *et al.* 2014; Eytan *et al.* 2015). There has been no research to our knowledge looking at the interrelationships between armed conflict and psychosis in Rwanda; indeed, there has not been significant published research regarding psychosis in any context in the country before.

Early treatment of schizophrenia is linked to better outcomes (Marshall *et al.* 2005) and accurately identifying individuals before illness onset holds promise for prevention (Mrazek & Haggerty, 1994; McGorry & Killackey, 2002). This is especially relevant in sub-Saharan Africa, where resources for managing psychotic disorders are extremely limited (Patel *et al.* 2006; Saxena *et al.* 2006; Ndetei *et al.* 2007). Assessing clinical risk early has significantly advanced the possibility of indicated prevention of full-blown psychotic disorders (Yung *et al*. 1996, 2004; Miller *et al*. 1999). Our group has conducted multiple studies in Kenya, investigating the prevalence of PLEs and risk for developing illness using self-report questionnaires (Mamah *et al*. 2012, 2013; Ndetei *et al*. 2012; Owoso *et al*. 2014). The Washington Early Recognition Center Affectivity and Psychosis (WERCAP) Screen (Mamah *et al.* 2014) was developed to overcome some of the challenges in assessing risk of developing psychotic disorders, guided by the existing literature on risk assessment and our previous efforts in Africa and the USA. Using the instrument, the primary aims of our study were to: (1) compare psychotic and affective risk symptoms between adolescents in Rwanda and Kenya – a geographically close country without the similar recent history of genocide, civil war or being directly affected by the Rwandan conflict – and (2) compare risk symptoms in Rwandan youth born before and after the war.

## Methods

### Participants

A total of 2800 Kenyan secondary school students from 10th through 12th grades (ages 14 and older) were recruited in September and October, 2013. These students came from a relatively rural area with families present there for generations and no known history of organised mass violence. In Rwanda, 1628 secondary school students from the final three grades (ages 14 and older) were recruited from ten secondary schools in Kigali and surrounding areas. A total of 627 Rwandan college students aged 19 and older were recruited from the former Kigali Health Institute (now part of the University of Rwanda, College of Medicine and Health Sciences) as well. Rwandan participants were recruited in February, 2014. All assessments were performed in the classroom and individually administered; participation was voluntary with all questions verbally answered before consent was signed and the study begun. The study was approved by the Institutional Review Boards (IRBs) of Washington University School of Medicine (no. 201306026), the Kenyatta National Hospital Ethics and Research Committee (no. KNH-ERC/A/137) and the Kigali Health Institute (no. KHI/IRB/21/2013).

### Assessment

All participants were administered the WERCAP Screen – an instrument which we have described previously (Mamah *et al.* 2014) and used in both the USA and Kenya (Mamah *et al.* 2016). The instrument is divided into two sections, one that estimates psychosis risk (pWERCAP) and the other the risk for developing bipolar disorder (termed ‘affectivity’-aWERCAP). The WERCAP Screen (psychosis portion) has previously been validated in a US sample against the SIPS (Structured Interview for Psychosis-Risk Syndromes) – an interview that utilises five psychotic-risk symptom subscales (Miller *et al.* 2003); a pWERCAP score >30 was consistent with meeting threshold for psychosis-risk state (sensitivity = 0.89; specificity = 1.0) (Mamah *et al.* 2014). The WERCAP consists of 16 question items: the first eight assess bipolar-risk and the latter eight psychosis-risk. Examples of screen questions are provided in a previous study (Mamah *et al.* 2014). The severity of symptom items is quantified by rating their frequency of occurrence and, if symptoms are present, their effect on functioning *at home*, *work or school, or with other people*. Options for frequency are ‘*none*’, ‘*once*’, ‘*rarely*’ (<yearly), ‘*sometimes*’ (>yearly-monthly), ‘*often*’ (>monthly-weekly) or ‘*almost always*’ (>weekly-daily). Five items in the affectivity section do not have an option for rating symptom effects on functioning, as estimating functionality could not be done accurately or they were considered to not be typically associated with readily identifiable dysfunction. Four options for rating functionality are provided: ‘*not at all*’, ‘*a little*’, ‘*moderately*’ and ‘*severely*’. Total scores are generated by a sum of frequency ratings (0–5) and functionality ratings (0–3) to give a measure of total risk symptom burden. Thus, the maximum score is 49 for the aWERCAP and 64 for the pWERCAP.

For the Rwandan participants, the WERCAP Screen was translated to *Kinyarwandan*, the language universally spoken and understood in Rwanda. Translation involved initial consensus by three Rwandan researchers fluent in English and *Kinyarwandan*, followed by a back-translation by an independent research assistant also fluent in both languages who had no knowledge of the original questionnaire's content. There were no issues with understanding of the translated version among Rwandan students. The original version of the instrument was used in Kenya, where English is well understood and used in schools.

### Data analysis

General statistical analyses were performed with IBM SPSS Statistics, Version 22.0 (IBM Corp., Armonk, NY). Students were compared using analysis of covariance (ANCOVA) and multivariate analysis of covariance (MANCOVA), controlled for age. For multivariate analyses, individual item scores (i.e. frequency + functionality, when applicable) on either the aWERCAP or pWERCAP were used as the dependent variable. *Post hoc* univariate analyses of aWERCAP or pWERCAP items following respective MANCOVAs were done using Bonferroni correction, with significance set at *p* < 0.0063 (i.e. 0.05/8). Chi-square (*χ*^2^) analysis assessed differences in symptom prevalence frequencies among the four groups outlined in the aims above.

## Results

### Demographic information

A Levene's test showed equality of variances (*p* > 0.05) across the four groups (Rwandans born pre-conflict, Rwandans born post-conflict (after 1994), Rwandan secondary school students and Kenyan secondary school students). Demographic information of the groups is displayed in Table 1. Among the 2800 Kenyans recruited, 2737 reported their gender. Of these, 1467 (53.6%) were female and 1270 (46.4%) were male. Among Rwandans, 1568 reported gender, with 672 (42.9%) females and 896 (57.1%) males. For Rwandan students born in or before 1994, 36.6% of those reporting gender were female with 63.4% male; Rwandans born after 1994 had an even gender report of 50% for both genders.

**Table 1.** Demographic and symptom table

Characteristics	Rwandan Sec. Sch.	Kenyan Sec. Sch.	Rwandans (pre/in-1994)	Rwandans (post-1994)	^a^*t*/*χ*^2^	^a^*p* ^a^CID	^b^*t*/*χ*^2^	^b^*p* ^b^CID
		(*n* = 1628)	(*n* = 2800)	(*n* = 1383)	(*n* = 784)			
Age – Total	18.8 (2.4)	16.3 (1.4)	21.9 (3.4)	17.0 (1.1)	37.3	<0.001	38.7	<0.001
Female	18.4 (2.2)	16.1 (1.3)	21.5 (3.0)	17.0 (1.2)	24.9	<0.001	31.0	<0.001
Male	19.1 (2.5)	16.6 (1.5)	22.1 (3.6)	17.1 (1.1)	26.6	<0.001	37.1	<0.001
Gender – *N* (%)					46.0	<0.001	36.1	<0.001
Female	672 (42.9)	1467 (53.6)	495 (36.6)	383 (50.0)				
Male	896 (57.1)	1270 (46.4)	857 (63.4)	383 (50.0)				
Unknown	60	63	31	18				
Affectivity – Total	10.47 (8.87)	8.52 (7.97)	10.47 (8.91)	8.61 (7.96)	7.3	<0.001 (1.44–2.47)	4.8	<0.001 (1.11–2.61)
Female	11.78 (9.13)	8.99 (8.15)	11.99 (9.11)	9.69 (8.52)	6.8	<0.001 (1.98–3.60)	3.9	<0.001 (1.12–3.47)
Male	9.50 (8.61)	7.92 (7.69)	9.53 (8.68)	7.53 (7.29)	4.5	<0.001 (0.87–2.28)	4.2	<0.001 (1.06–2.93)
Psychosis – Total	8.61 (10.9)	8.21 (10.3)	8.50 (10.90)	6.87 (9.54)	1.1	0.279 (−0.29 to 1.00)	3.5	<0.001 (0.75–2.51)
Female	9.75 (11.5)	8.23 (10.3)	9.32 (11.17)	8.16 (10.98)	2.9	0.004 (0.50–2.54)	1.5	0.127 (−0.33 to 2.63)
Male	7.82 (10.4)	8.17 (10.1)	8.04 (10.74)	5.66 (7.83)	−0.78	0.436 (−1.23 to 0.53)	4.4	<0.001 (1.31–3.45)

a Comparison between Rwanda and Kenya secondary school students.

b Comparison between pre/in-1994 and post-1994 born Rwandan students.

Age, aWERCAP and pWERCAP scores are given in means (standard deviation). Gender is given in absolute numbers (percentages).

CID = 95% Confidence Interval of the difference in scores.

Affectivity and Psychosis scores were derived from the total scores from affectivity (#1–8) and psychosis (#9–16) items on the WERCAP Screen.

### WERCAP score comparisons

#### Older *v*. Younger Rwandan Students

**Fig. 1.**
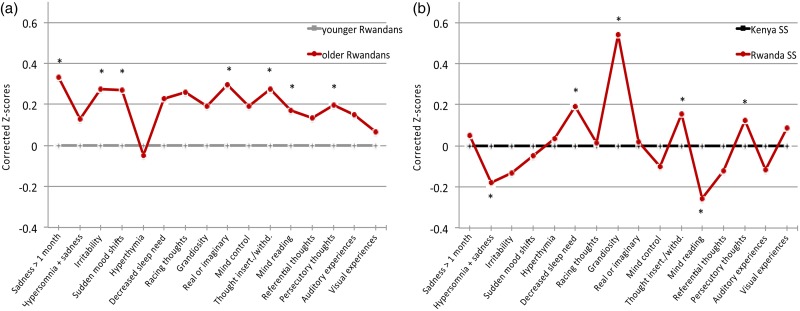
Symptom scores in Rwandan and Kenyan adolescents. The graph shows corrected mean z-scores of total scores from each item on the WERCAP, i.e. frequency of occurrence + functionality (when applicable). In (*a*), *z*-scores in older Rwandan students were corrected for age and normalised against the younger Rwandan group. In (*b*), *z*-scores for Rwandan secondary school students were corrected for age and normalised against the Kenyan students. The first eight questions probe affective symptoms; the last eight questions probe psychotic symptoms. Significant differences after Bonferroni correction (*p* < 0.003) are indicated with asterisks.

Rwandan students born in or before 1994 had significantly higher total psychosis (*p* < 0.001) and affectivity (*p* < 0.001) scores compared with Rwandan students born later (Table 1). Older Rwandan males had higher aWERCAP and pWERCAP scores than younger males (*p*’s < 0.001). However, while older Rwandan females had higher aWERCAP scores than younger females (*p* < 0.001), there was no significant pWERCAP difference seen in females. Age-corrected *z*-scores from univariate analyses for individual WERCAP items are shown in Fig. 1*a*; older Rwandans had significantly higher scores on multiple items across both affective and psychotic domains. For older students, 5.35% met the positive pWERCAP threshold while only 3.19% of the younger Rwandan cohort did – a statistically significant finding (*χ*^2^ = 5.36; *p* = 0.02).

#### Kenyan *v*. Rwandan Secondary Schoolers

Table 1 shows the mean total scores across Rwandan and Kenyan students. Total pWERCAP and males’ pWERCAP scores did not differ between countries; differences in pWERCAP in females did not remain after age correction. Similarly, any differences in aWERCAP scores between Kenyan and Rwandan secondary schoolers did not remain after controlling for age.

We also performed MANCOVAs to allow for the between-country comparison of affective and psychosis items *as a group* as a way to more finely detect differences. A MANCOVA using the eight affective items as dependent variables resulted in a significant difference between Rwandan and Kenyan secondary schoolers (Wilks's *λ* = 0.905; *p* < 0.001). Age-corrected affective item *z*-scores are shown in Fig. 1*b*. *Post hoc* univariate analyses showed significant group effects for ‘*hypersomnia and sadness*’ (*p* < 0.001), ‘*decreased need for sleep*’ (*p* < 0.001) and ‘*grandiosity*’ (*p* < 0.001). The largest effect on overall MANCOVA (as determined by observed power and effect size in the corrected model) was for ‘*grandiosity*’, followed by ‘*decreased need for sleep*’ – both of which were significantly higher in Rwandan compared with Kenyan secondary schoolers.

Using psychosis-risk items, a MANCOVA here also revealed a significant group effect between Rwandan and Kenyan students (Wilks's *λ* = 0.948; *p* < 0.001). Age-corrected *z*-scores are shown in Fig. 1*b*. *Post hoc* analyses showed significant differences in the items ‘*thought insertion/withdrawal*’ (*p* < 0.001), ‘*mind reading*’ (*p* < 0.001) and ‘*persecutory thoughts*’ (*p* < 0.001); there was trend towards significance for ‘*visual experiences*’ (*p* = 0.013). All items except for ‘*mind reading*’ were significantly higher in Rwandan students.

The *χ*^2^ analysis showed no significant difference between countries for secondary schoolers meeting threshold for psychosis-risk state (5.22% – Rwanda, 4.6% – Kenya).

### Symptom prevalence

**Fig. 2.**
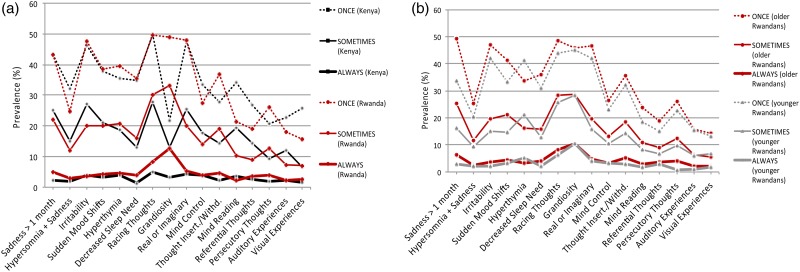
Prevalence of affective and psychotic symptoms. The figure depicts the percentage of students that endorsed each symptom on the WERCAP Screen at least once, sometimes or almost always. (*a*) Comparison of prevalence in Rwandan and Kenyan secondary school students. (*b*) Comparison of prevalence in Rwandan students born before (‘older’) and after (‘younger’) Rwandan genocide and end of Civil War in 1994. The first eight questions probe affective symptoms; the last eight questions probe psychotic symptoms.

Rwandan secondary school students had substantially higher ‘*grandiosity*’ prevalence than their Kenyan counterparts, at all occurrence frequencies (Fig. 2*a*). This ranged from 49.1% (Rwanda) *v*. 21.9% (Kenya) experiencing grandiosity at least once (*χ*^2^ = 350.3; *p* < 0.001), to 12.7% (Rwanda) *v*. 3.3% (Kenya) reporting grandiosity almost always (*χ*^2^ = 145.4; *p* < 0.001). Other affective and psychotic symptoms showed lesser prevalence differences across countries. Pre-1994 and post-1994 birth Rwandans had differences in various symptom frequency prevalences; the most significant difference between groups was for ‘*sadness for at least one month*’. Interestingly, both birth cohorts in Rwanda showed virtually the same prevalence for the grandiosity item across all frequencies (Fig. 2*b*).

### Frequency of symptom occurrence

**Fig. 3.**
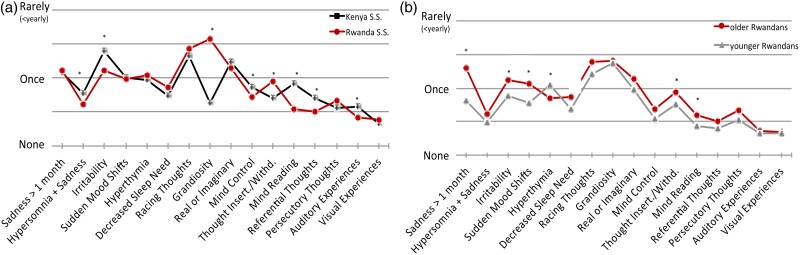
Mean symptom occurrence frequencies. The graph shows corrected mean occurrence frequencies from each item on the WERCAP. Options for occurrence frequencies included: *none, once, rarely, sometimes, often* and *almost always*. (*a*) Comparison in Rwandan and Kenyan secondary school students. (*b*) Comparison in older and younger Rwandan students. First eight questions probe affective symptoms; last eight questions probe psychotic symptoms. Significant differences after Bonferroni correction (*p* < 0.003) are indicated with asterisks.

Analyses of affective symptom frequencies found multiple items different between countries (Fig. 3*a*). Most prominently, mean ‘*grandiosity*’ frequency was substantially higher in Rwandans compared with Kenyans (*p* < 0.001). In comparing older and younger Rwandans (Fig. 3*b*), many symptoms showed older Rwandans experiencing them more often.

### Functional impact of symptoms

**Fig. 4.**
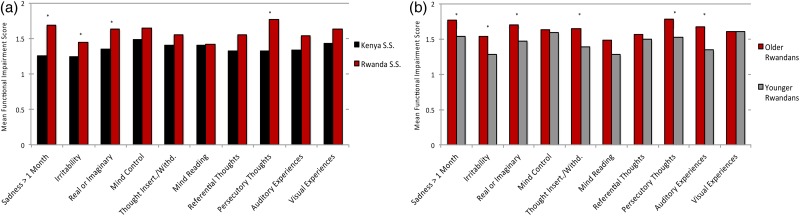
Functional impairment from endorsed symptoms. Bar graphs show mean scores on functionality in individuals who endorsed having experienced the specified symptoms at least once. Functionality is assessed in ten items on the WERCAP Screen, and is rated as one of: *not at all, a little, moderately* or *severely*. (*a*) Comparison in Rwandan and Kenyan secondary school students. (*b*) Comparison in older and younger Rwandan students. Significant differences after Bonferroni correction (*p* < 0.005) are indicated with asterisks.

After Bonferroni correction (*p* < 0.005), Rwandan secondary school students who reported symptoms had significantly higher functional impairment compared with Kenyans on the items ‘*sadness*’, ‘*irritability*’, ‘*real v. imaginary*’ and ‘*persecutory thoughts*’ (Fig. 4*a*). Older Rwandans showed higher impact on functioning than younger Rwandans in six items: ‘*sadness*’, ‘*irritability*’, ‘*real v. imaginary*’, ‘*thought insertion/withdrawal*’, ‘*persecutory thoughts*’ and ‘*auditory experiences*’ (Fig. 4*b*).

### Gender effects

Mean aWERCAP scores in Kenyan female secondary schoolers were higher than in males (f: 8.99[8.15]; m: 7.92[7.69]; *p* < 0.001). Mean pWERCAP scores did not show gender differences (f: 8.23[10.3]; m: 8.17[10.1]; *p* = 0.874). Female students endorsed higher average frequencies of several affective symptoms, although only ‘*irritability*’ showed a statistically significant difference after correction for multiple comparisons (*p* < 0.003). There was no gender difference functional impairment from symptoms among Kenyans.

Among Rwandan secondary school students, females had higher aWERCAP scores than males (f: 11.78[9.13]; m: 9.50[8.61]; *p* < 0.001). Females had higher pWERCAP scores as well (f: 9.75[11.5]; m: 7.82[10.4]; *p* = 0.001). Females endorsed higher symptom frequencies across both affective and psychosis domains compared to males. After Bonferroni correction (*p* < 0.003), significant differences were observed for ‘*sadness*’, ‘*hypersomnia + sadness*’, ‘*irritability*’, ‘*real v. imaginary*’, and ‘*thought insertion/withdrawal*’ – all of which were more frequent in Rwandan females than males. There were no gender differences in functional impairment from symptoms.

### Other analyses

We compared youth born after 1994 in both countries to investigate potential effects of country or post-conflict environment on a population without the direct exposure to the genocide or events of the war. For the aWERCAP, young secondary school students in Kenya and Rwanda did not differ in scores; pWERCAP scores, however, were lower in the younger Rwandan population (Kenya: 8.26[10.2]; Rwanda: 6.88[9.55]; *p* < 0.001). For secondary schoolers born before 1994, Rwandans scored higher on the aWERCAP (Rwanda: 12.2[9.26]; Kenya: 10.0[8.23]). When comparing across gender, young Rwandan males continued to demonstrate lower pWERCAP scores compared with Kenyans (Kenya: 8.20[10.1]; Rwanda: 5.66[7.83]; *p* < 0.001) while young females in both countries showed no difference in the pWERCAP (Kenya: 8.28[10.3]; Rwanda: 8.19[11.0]). While young Kenyans showed no difference between genders in the pWERCAP, young Rwandan females had higher scores compared with Rwandan males (*p* < 0.001). For young Rwandans, significantly more females met the high-risk threshold on pWERCAP compared with males (4.96% *v*. 1.57%; *χ*^2^ = 6.99; *p* = 0.008); no such gender difference was seen in young Kenyans.

## Discussion

The current study is the first to our knowledge that investigates psychosis-risk or bipolar symptoms in Rwanda. The large sample size (over 5000) provides rich information about the extent of psychotic and affective symptoms. Our findings expand knowledge of war-related psychopathology by investigating a region lacking in research on the subject and by examining youth born after conflict in a country as opposed to only those who lived through conflict. Our study design allowed for both the comparison of Rwandan secondary school students to a similarly aged population in a nearby country as well as an intra-country comparison of Rwandan students born both before and after the genocide and end of civil war. There were several notable findings in our investigation.

### Older Rwandans have higher symptom risk and functional impairment than younger Rwandans

Across both symptom domains, Rwandans born in and before 1994 had higher scores than Rwandans born afterwards. Functional impairment was also higher across multiple items and there were significantly more older Rwandans meeting criteria for psychosis-risk state compared with the younger cohort. These differences persisted after controlling for age. As additional evidence of the high likelihood of time of birth affecting symptoms, when comparing Kenyan students born in and before 1994 *v*. Kenyans born after, there is no difference in psychosis, affective, or functional symptoms after controlling for age. Different symptoms among the Rwandan age cohorts are likely secondary to effects of being alive during the genocide and war as opposed to a simple effect of age. Even though the older cohort was on average less than 2 years old by the war's end and lived through the same post-conflict environment as their younger counterpart, birth before the war's end appears a critical factor in risk. As there are relatively few studies globally looking at the influence of conflict and war on psychotic risk in youth, our findings help to elucidate these putative effects. However, as psychosis-risk determinations have not yet been shown to consistently predict later development of full psychotic diagnoses to satisfactory degree (Yung *et al.* 2004; Cannon *et al.* 2008; Ruhrmann *et al.* 2010), the full impact of our findings would be further supported with longitudinal follow-up of individuals. Nevertheless, the increased psychotic symptoms and functional impairment – especially in older Rwandan students – are important signs of distress from a public health perspective whether or not there is future development of a diagnosed disorder.

### Rwandan students have higher symptom risk scores than Kenyans

Age-corrected multivariate analyses showed multiple affective and psychotic symptoms with higher scores among Rwandans compared with Kenyans, a finding consistent with past studies in conflict areas (Catani *et al.* 2009; Priebe *et al.* 2010*a*; Amone-P'Olak *et al.* 2014), though lack of between-country difference on initial summary WERCAP scores suggests caution in over-interpretation of result. However, a very salient symptom with significant group differences in prevalence and frequency was *grandiosity –* endorsed substantially more in Rwanda. The marked difference between countries may speak to something particular about this symptom in populations living after the cessation of significant conflict. Past research has suggested a development of relative optimism living through the trauma of ongoing conflict in Israel (Bleich *et al.* 2005). An additional factor may be a difference in the ability to appreciate one's existing life situation in the context of a recent ending of societal violence. A study of former American prisoners of war during the Vietnam War showed that the ability to experience post-traumatic growth and obtain a positive outlook after traumatic experiences strongly correlated with appreciation of life (Feder *et al.* 2008). This would suggest that grandiosity in these individuals is not necessarily related to psychopathology or the bipolar-risk state, which the WERCAP Screen was designed to measure. That being said, the grandiosity item on the WERCAP probes for the feeling of having ‘great abilities or supernatural powers’ not shared by anyone else in the world. Longitudinal studies of individuals with high grandiosity scores would be needed to determine eventual clinical outcomes.

Of note, when summary WERCAP scores of secondary school students born post-conflict across countries were compared, Rwandans showed no difference in affective scores and had lower pWERCAP scores compared with post-1994 birth Kenyans. This suggests that while Rwandan secondary schoolers show more risk symptomatology than Kenyans, much of this may be explained by the older Rwandans born before the end of the war and genocide.

### Symptoms cause more dysfunction in Rwandan than Kenyan adolescents

Among individuals who reported symptoms, Rwandan secondary school students were more functionally impaired from symptoms than their Kenyan counterparts. The reason for this difference is unclear, and may be related to some adverse impacts of a post-war climate in Rwanda. Research on individuals with early psychosis syndromes found greater functional impairment in those who experienced abuse early in life compared with those who experienced none (Alameda *et al.* 2015). Similar results were seen in a 2010 study of correlates to functional outcome among first-episode psychotic patients (Conus *et al.* 2010); other studies have shown significant associations between early-life trauma and symptom intensity in bipolar disorder, depression and anxiety (Martins *et al.* 2014; Duhig *et al.* 2015). Indeed, the extreme stress caused by the aftermath of war and genocide – particularly in children – can help explain much of the risk for psychosis in this vulnerable population (Veling *et al.* 2016). Neuroendocrine findings on the relationship between environmental stress, cortisol and psychosis risk also provide some clues on potential mechanisms for dysfunction development (Carol & Mittal, 2015). These findings support the possibility that growing up in a country laden with the aftermath of war would increase the magnitude of dysfunction from psychiatric symptoms.

### Rwandan female students report greater psychosis than males

Both Rwandan and Kenyan female secondary schoolers had higher aWERCAP scores than males. However, only the Rwandan secondary school females had increased psychosis severity compared with their male counterparts. Younger (post-1994 birth) Rwandan females also showed more psychosis-risk symptoms than young males (a finding that was not seen with young Kenyans). Additionally, Rwandan females did not show diminution in psychotic symptoms from the older to younger population (as was seen with Rwandan males). Furthermore, significantly more young Rwandan females than males met threshold for psychosis high-risk, while no such gender differences were seen in the young Kenyan sample. Importantly, these gender differences in Rwanda held true for youth as a whole as well as for those born after 1994. While males are known to generally experience more severe psychotic symptoms with average onset several years before females (Loranger, 1984), our findings show that Rwandan students reveal the opposite pattern. This suggests the possibility of an effect of country or post-conflict environment that has differential gender effects. Recent research has shown associations between early experiences of trauma and core features of psychotic illness that are stronger in females than males (Haug *et al.* 2015) as well as the role of stress sensitivity in mediating the relationship between early life traumas and later psychotic symptoms – a phenomenon that has been seen to occur in females as opposed to males (Gibson *et al.* 2014). Future longitudinal outcome studies would be helpful to better contextualise the meaning of findings in our investigation.

### Limitations

Due to the nature of our study as an examination of the general youth population, we did not have exclusions other than age and school attendance. Our study's setting and logistics precluded us from collecting additional demographic variables such as previous refugee status amongst Kenyans (although the location in Kenya makes such status unlikely), any previous psychiatric history, or drug use. While Kenya is without a recent history of war and genocide, the countries may have other unaccounted for cultural differences that may explain some of the differences seen in symptom report. Additionally, the language used in the administered instrument was different between countries, which could have led to slightly different interpretations of questions despite our diligent efforts during translation to maintain the integrity of the original questionnaire. The *grandiosity* item in particular may have had different interpretations depending on language and phrasing (Mamah *et al.* 2012; Ndetei *et al.* 2012). However, in a separate analysis *grandiosity* was also more prevalent and had higher scores in Rwandan secondary schoolers compared with Rwandan college students. This suggests that language and cultural differences cannot account for all disparities observed between countries.

While the use of a self-report screen has some advantages over a structured interview in terms of administration time and in obtaining potentially sensitive information (Bowling, 2005), the ability to accurately interpret a participant's response or to ensure full understanding of an item is less. Additionally, long-term follow-up of individuals would be needed to determine the rate of progression to diagnosable psychopathology, something that cannot be completed with a cross-sectional study. Despite these limitations, this study represents important first steps into wider investigations of psychosis and affective risk symptoms in a part of the world where such research is lacking. An investigative strategy that addresses potential risk burden in a general population exposed to serious conflict has important policy and health provision implications in a non-resource-rich country. Broader and more longitudinal studies along with further work into connections between serious trauma and subsequent development of mental illness – particularly psychosis – will provide important contributions to our knowledge on psychiatric risk in young and vulnerable populations around the world.

## Availability of data and materials

The data from our study are not publicly available as data sharing of collected information was not part of the approved IRB documentation across the three review bodies nor was it part of the consent materials provided to participants.
